# A Study on Domestic Accidents Among Women in a Coastal Area of Mangaluru, Karnataka

**DOI:** 10.7759/cureus.30605

**Published:** 2022-10-23

**Authors:** Shubhankar Adhikari, Imaad Mohammed Ismail

**Affiliations:** 1 Community Medicine, Yenepoya Medical College, Mangaluru, IND

**Keywords:** homemaker, burn, rural community, injuries, domestic accidents

## Abstract

Background

Domestic accidents are a worldwide health problem because of an epidemiological transition from communicable to non-communicable diseases. ‘Modern Day Epidemic’ is the term coined for accidents. Due in large part to the fact that they have not been accurately counted, domestic accidents have not yet received the same level of attention as traffic accidents or work-related injuries. The objectives of the study were (i) to assess the prevalence of domestic accidents among women, and (ii) to study the epidemiological factors associated with domestic accidents.

Materials and methods

This was a community-based cross-sectional study carried out in Panemangaluru, Dakshina Kannada, from Jun 2016 to December 2017. Complete enumeration was done and all the women above 18 years of age were interviewed. Information on sociodemographic characteristics, a history of home accidents, and accident-related factors were gathered using a pre-tested, validated, semi-structured questionnaire. Statistical analysis was done using the Statistical Package for Social Sciences (SPSS) version 22 (IBM Corp., Armonk, NY, USA).

Results

Among 1573 subjects, the prevalence of domestic accidents among rural women was 35.9%. Most (28.5%) domestic accidents were in the age group of 30 to 39 years. The most common domestic accident was injuries from sharp objects (51.2%). The majority of domestic accidents (38.9%) occurred in the morning or evening hours, and the kitchen (75.9%) was the most common place of occurrence. The majority of victims received care at home, and in 96.2% of domestic accidents, complete recovery was noted.

Conclusion

The prevalence of domestic accidents in rural women was 35.9%. Injuries from sharp objects were the most common type of domestic accident.

## Introduction

Accidents can occur in a wide variety of environments and there is a possibility of accidents in every sphere of human life, i.e. at home, while traveling, at play, and work [1,2]. In the modern world, danger prevails not only on the roads but also in the home as well as on playgrounds [2,3]. Home is the most likely location for unintentional injuries [4].

A domestic accident is an accident that takes place at home or in its immediate surroundings. Generally, all accidents are not connected to traffic, vehicles, or sports, which brings a varying measure of distress to the victim and family members [2,5]. The consequences may be disastrous both for the individual and the society when the accident results in permanent disability, which leads to loss of earning power and may not able to enjoy a normal productive life [6].

Unintentional injuries are one of the five most common causes of death worldwide, a significant contributor to disability, and a major cause of the potential loss of life. About 5.8 million people die of injuries worldwide each year and it causes 16% of the global disease burden. Injuries account for one-seventh of healthy life years lost in the world. It is estimated that by 2020 they will account for one in five, with low and middle-income countries bearing the brunt of this increase, and the problem is more prominent in the rural area [7-8].

Against the above backdrop, the present study was conceived and conducted to assess the prevalence and the epidemiological factors associated with domestic accidents in a rural community. The result of the study may aid health planners in developing appropriate preventive measures.

## Materials and methods

Study setting

Dakshina Kannada district is situated in the coastal belt of Karnataka, India, and Mangaluru city is its administrative headquarters [9]. The Institute where the study was done (AJ Institute of Medical Sciences and Research Center) has four rural field practice areas, namely Sajipa, Mani, Panemangaluru, and Bantwal. From these areas, Panemangaluru was selected randomly using the lottery method, which is 25 km away from Mangaluru city.

Inclusion and exclusion criteria

All women over 18 years of age involved in household work (e.g., cooking, cleaning, washing, and ironing clothes) were included. Exclusion criteria included those households found locked (even after two consecutive visits) and non-response of the participants.

Data collection and analysis

Panemangaluru has six sub-areas: Bolangadi, Dasaragudde, Nehrunagar, Melkar, Marnabail, and Nandavara, consisting of a total population of 1867 women aged more than 18 years, according to a survey done by Rural Health Training Centre in 2015. The participant’s consent form was prepared in the local language and consent was taken after explaining the purpose of the study to the participants. Complete enumeration was done and all the women over 18 years of age were interviewed using a pre-tested semi-structured validated questionnaire. Information regarding the history of domestic accidents, socio-demographic parameters, and their working environment during the past year was collected in their local language. After collecting data, those with a history of domestic accidents that required treatment were referred to the health care center. The data were entered in Microsoft Excel version 14 (Microsoft Corp., Redmond, WA, USA), compiled, and analyzed by using Statistical Package for Social Sciences (SPSS) version 22 (IBM Corp., Armonk, NY, USA). Descriptive statistics like mean, standard deviation, and inferential statistics such as the chi-square test were used to analyze the data. The Institutional Ethics Committee approved the study protocol (AJEC/REV/56/2015-16).

## Results

In the present study it was observed that out of the 1573 study population, the majority i.e., 448 (28.5%) were in the age group of 30 to 39 years, followed by 302 (19.2%) who were in the age group of 40 to 49 years. The median age of females was 41.76 ± 13.38 years (Table 1).

**Table 1:** Socio-demographic characteristics of the study population (N=1573) * P-value < 0.05 statistically significant, H/O: History of

	H/O Domestic accident		Total	Chi-square	P-value*
	Yes (%)	No (%)			
Age					
< 20	10 (31.3)	22 (68.8)	32	54.61	< 0.01
20-29	81 (27.6)	213 (72.4)	294		
30-39	199 (44.4)	249 (55.6)	448		
40-49	130 (43)	172 (57)	302		
50-59	72 (23.9)	229 (76.1)	301		
> 60	54 (27.6)	142 (72.4)	196		
Education					
Illiterate	38 (31.1)	84 (68.9)	122	23.10	< 0.01
Primary school	155 (32.2)	326 (67.8)	481		
Higher primary school	135 (39.7)	205 (60.3)	340		
High school	110 (34.1)	213 (65.9)	323		
PUC/Diploma	84 (44)	107 (56)	191		
Degree	24 (20.7)	92 (79.3)	116		
Occupation					
House-wife	331 (35.7)	597 (64.3)	928	2.71	> 0.05
Skilled	29 (33.7)	57 (66.3)	86		
Semi-skilled	8 (22.9)	27 (77.1)	35		
Un-skilled	178 (34)	346 (66)	524		
Type of family					
Nuclear family	207 (36.8%)	356 (63.2%)	563	3.69	> 0.05
Joint family	282 (32.7%)	581 (67.3%)	863		
3 Generation family	57 (38.8%)	90 (61.2%)	147		

Of the women majority, 481 (30.6%) have primary level education, 340 (21.6%) attended education till higher primary, 116 (7.4%) completed a Bachelor's degree, 928 (59%) are homemakers, 875 (55.6%) belong to the Muslim community, 595 (37.8%) are Hindus, and a majority of them belong to the upper-middle and lower-middle class.

In this present study, the prevalence of domestic accident cases is 34.7% and the majority of episodes of domestic accidents is 35.9% between June 2016 and December 2017. Injuries from sharp or pointed instruments (51.2%) were the most common type of domestic accident followed by, burns (24.9%), falls (22.7%), and electrical injuries (1.2%). There were no cases of poisoning and drowning during the study period and the findings are statistically significant (Table 2).

**Table 2:** Distribution of domestic accidents among the study population (N=1573) * Multiple episodes of accidents, H/O: History of

Domestic accident	Frequency (%)
H/o domestic accidents	
Cases with a single episode	527 (33.5)
Cases with multiple episodes	19 (1.2)
Total	546 (34.7)
Type of domestic accident	
Injuries from sharp or pointed instruments	290 (51.2)
Burns	141 (24.9)
Fall	128 (22.7)
Electrical injuries	6 (1.2)
Total	565*

The most common place for accidents was in the kitchen (75.9%) and the upper limb was involved 78.6% times. The majority of these accidents happened during the evening time and 90.1% of women took care of their injuries at home. Complete recovery was seen in 96.2% of women who had an injury and the findings are statistically significant (Table 3).

**Table 3:** Association of epidemiological factors of domestic accidents among the study population (N=1573) * P-value < 0.05 statistically significant

	Fall (%)	Burn (%)	Injuries from sharp objects (%)	Electric shock (%)	Total (%)	Chi-square value	P-Value*
Place of accident							
Kitchen	107 (20.3)	113 (21.5)	256 (48.6)	0 (0)	475 (90.3)	158.468	< 0.01
Bathroom	3 (0.6)	5 (0.9)	2 (0.4)	0 (0)	10 (1.9)		
Living room	5 (0.9)	8 (1.5)	0 (0)	4 (0.8)	17 (3.2)		
Immediate surrounding	0 (0)	5 (0.9)	16 (3)	0 (0)	21 (4)		
Terrace	3 (0.6)	0 (0)	0 (0)	0 (0)	3 (0.6)		
Time of accident							
Morning	37 (7)	40 (7.6)	119 (22.6)	4 (0.8)	200 (38)	39.810	< 0.01
Afternoon	17 (3.2)	39 (7.4)	45 (8.5)	0 (0)	101 (19.1)		
Evening	58 (11)	41 (7.8)	106 (20.1)	0 (0)	205 (38.9)		
Night	6 (1.1)	1 (2.1)	4 (0.8)	0 (0)	21 (4)		
Body parts involved							
Head and neck	0 (0)	7 (1.3)	0 (0)	0 (0)	7 (1.3)	315.886	< 0.01
Back	28 (5.3)	0 (0)	0 (0)	0 (0)	28 (5.3)		
Upper limb	32 (6.1)	117 (22.2)	263 (49.9)	4 (0.8)	416 (79)		
Lower limb	58 (11)	7 (1.3)	11 (2.1)	0 (0)	71 (14.4)		
Place of Treatment							
Home care	108 (20.5)	109 (20.7)	251 (47.6)	0 (0)	468 (88.8)	262.353	< 0.01
OPD care (Govt.)	2 (0.4)	7 (1.3)	6 (1.1)	0 (0)	15 (2.8)		
OPD care (Private)	5 (0.9)	13 (2.5)	8 (1.5)	0 (0)	26 (4.9)		
In-patient care (Govt.)	0 (0)	0 (0)	6 (1.6)	0 (0)	6 (1.1)		
In-patient care (Private)	3 (0.6)	0 (0)	0 (0)	0 (0)	3 (0.6)		
No treatment	0 (0)	2 (0.4)	3 (0.6)	4 (0.8)	9 (1.8)		
Tetanus toxoid							
Not received	112 (21.3)	119 (22.6)	246 (46.7)	4 (0.8)	481 (91.3)	3.144	0.370
Received	6 (1.1)	12 (2.3)	28 (5.3)	0 (0)	46 (8.7)		

## Discussion

The pattern of domestic accidents is complex, involving cultural, social, and economic factors [2,10]. The host factors (age, sex, place of residence, co-morbidity, alcohol and drug use, etc.), agent factors (several domestic products that people frequently use for day-to-day activities), and environmental factors frequently determine the type of domestic injury (type of housing, flooring, roofing, safety environment, etc.) [2,11].

Every home accident causes the victim and the family members varied degrees of distress. When an accident leaves a victim permanently disabled and renders them unable to work or live a regular, productive life, the results could be terrible for both the person and society [6].

This study was undertaken to understand the prevalence of domestic accidents in adult women residing in a rural community and determine the associated factors. In the present study, the prevalence of domestic accidents was found to be 35.9%. The current study prevalence is much higher than previously reported studies. George et al. observed a 10.5% prevalence of accidents for all age groups and both sex (66% of victims were women) in their study in rural Kerala [11]. Ramesh et al. observed that the overall prevalence of domestic accidents was 9.6% and domestic accidents were found to be common among women i.e., 66% [12]. Sudhir et al. observed that the overall prevalence of domestic accidents was 9.4% and 68.2% of accidents were observed among women [13]. Kommula et al., in their study, observed a 2% prevalence and that 53.9% of the accident victims were females [14]. Similarly, Masthi et al. observed a 13.3% prevalence of domestic accidents in females [12].

Prevalence of domestic accidents was found to be most common in the 30 to 49 years age group compared to other age groups and it was found to be statistically significant, and findings are comparable to the study done by Kommula et al. where domestic accidents ranged in the age group of 31 to 45 years (53.9%) [14]. In the present study, domestic accidents were found to be more common among housewives (35.7%), followed by un-skilled workers (34%), skilled workers (33.7%), and semi-skilled workers (22.9%). However, it was not found to be statistically significant. Similar results were found in a study by Sudhir et al., where domestic accidents were more common among housewives (26.6%) [13]. This may be due to them spending more time indoors.

Injuries from sharps objects (51.2 %) were found to be the most common domestic accidents, followed by burns (24.9 %), and falls (22.7%), while only 1.2% of women had a history of electric injury. In Masthi et al.'s study, the most common accidents reported were falls followed by injuries from sharp objects, and burns [12]. Similar results were also found by George et al. in their study [11]. According to a study done by Bhanderi et al., the most common accidents reported were falls, while other accidents noted were burns, scalds, electrocution, injuries, and accidental poisoning [8]. Chaurasia et al. observed a higher proportion of falls, burns, and scalds in their study [15].

The most common place of domestic accidents was the kitchen (75.9%), followed by immediate surroundings (11.7%), bathroom (7.3%), living room (4.1%), and terrace (1.1%). This can probably be attributed to the fact that while women are in the kitchen, they are exposed to different objects which may cause injuries such as a knife, peelers, etc. [2]. However, in a study by Aggarwal et al., 53.3% of the injuries occurred in the courtyard, followed by the living room (20.6%) and kitchen (16.3%) [16]. A study by Sudhir et al. observed that the most common place for accidents was the courtyard (49.7%) followed by the kitchen (15.8%) [13]. George et al. observed that the most common places of accidents were the courtyard and kitchen (37.7%), followed by the bedroom (13.6%) [11]. There is a difference in the place of occurrence of these domestic accidents because our study participants are women.

Most commonly, accidents occurred during morning and evening hours and the most frequent site of injuries was the upper limb. Similar results were observed in other studies [6,8,11]. However, Mukhopadhya, in his study, mentioned that a majority (40.1 %) of cases had lower limb injuries [17].

Around 90.1% of domestic injuries were taken care of at home itself and complete recovery was seen in most women, which is consistent with findings by Bhanderi et al. [8].

Limitation

The study may have recall bias as some participants might have missed recalling and reporting a domestic accident.

## Conclusions

The objective of the current study was to emphasize the epidemiological characteristics of domestic accidents among women in rural areas. According to the study, the prevalence of domestic accident cases and episodes was 34.7% and 35.9%, respectively. The kitchen was the most frequent location for domestic accidents, which mostly occurred in the morning and evening. When compared to other body parts, the upper limb was more actively involved. The explanation could be that women spend more time at home and are more actively involved in everyday household tasks in rural areas. Due to the high frequency of injuries from sharp objects and burns, proper planning of the house, adequate lighting, and raising awareness regarding prevention and use of first aid kit using information, education, and communication (IEC) material, may aid in lessening their occurrence.
